# Ara-C, Idarubicine and Gentuzumab Ozogamicin (AIM) as Salvage Treatment in Advanced Acute Myeloid Leukemia Patients

**DOI:** 10.4084/MJHID.2012.072

**Published:** 2012-11-06

**Authors:** Saveria Capria, Silvia Maria Trisolini, Clara Minotti, Caterina Stefanizzi, Luisa Cardarelli, Claudio Cartoni, Daniela Diverio, Maria Stefania De Propris, Marco Mancini, Alessandra Micozzi, Robin Foà, Giovanna Meloni

**Affiliations:** Hematology, Department of Cellular Biotechnologies and Hematology, “Sapienza” University of Rome, Rome, Italy

## Abstract

Long-term survival of relapsed/refractory acute myeloid leukemia (AML) remains a major problem, particularly in patients not eligible for transplantation.

We hereby evaluated the feasibility and efficacy of adding Gemtuzumab Ozogamicin to salvage chemotherapy (Ara-C, Idarubicine, Peg-Filgrastim) in relapsed/refractory AML. The main endpoints were: the rate of complete remissions (CR) and the proportion of patients capable of undergoing a stem cell transplant.

Fourty-two patients were enrolled. The overall CR rate was 76% and no induction deaths were reported. In 56% of patients, a transplant procedure could be performed. The treatment schedule proved feasible and well tolerated, providing a high CR rate and a useful bridge to transplant.

## Introduction

Patients with recurrent or refractory acute myeloid leukemia (AML) have a poor prognostic likelihood. Salvage regimens generally include high-dose Ara-C, but whenever a second CR is attained, the median duration of a second relapse-free interval (RFI) is considerably shorter than the first one, highlighting the need to develop novel therapies.^1^–^2^ In patients who are able to undergo an allogeneic hematopoietic stem cell transplant (HSCT), long-term survival rates range from 25% to 40%.^4^ However, only a minority of patients can benefit from a HSCT because of co-morbidities and treatment-related toxicities. Thus, the goal of novel therapies should be to achieve complete remissions (CR) with the lowest possible toxicities, in order to provide a bridge to transplant.^3^

Gentuzumab Ozogamicin (GO) is a conjugate of an anti-CD33 humanized monoclonal antibody and the anthracycline calicheamicin with an attractive mechanism of action, since the CD33 antigen/antibody complex is rapidly internalized in the leukemic cell prior to drug delivery.^4^ Pivotal studies of (GO) as single agent at 9 mg/m^2^ in relapsed AML have shown an overall response rate of 28% and encouraging results have been obtained in phase II trials in which it has been employed in combination with other cytotoxic agents.^5^–^8^

The EORTC/GIMEMA leukemia group tested in a phase II study (AML-15) the feasibility and safety of the sequential combination of GO and conventional chemotherapy in patients older than 60 years of age with untreated AML, confirming the feasibility and activity of such a treatment strategy in this group of patients.^9^

Moreover, several phase II trials have utilized the combination of Mylotarg with chemotherapy in AML patients with advanced disease, reporting a response rate between 12% and 42%, and a high treatment-related mortality.^10^–^12^ The main dose limiting toxicity of the compound is liver toxicity and the incidence of veno-occlusive disease; thus, when combined with chemotherapy, it is currently used at lower doses.^13^

In this retrospective analysis we evaluated the efficacy and safety of the administration of GO 3 mg/m^2^ after intensive chemotherapy consisting of intermediate doses of Ara-C and Idarubicine, and followed by Peg-filgrastim in adult patients with refractory/relapsed AML The main endpoints of the study were: the rate of second CR, the survival rate and the possibility of patients achieving a CR to undergo a transplant procedure.

## Patients and Methods

In this study we retrospectively analyzed the outcome of 42 adult patients with advanced phase AML consecutively treated in our Institute between 2005 and 2010. The main inclusion criteria were: diagnosis of AML with the exclusion of acute promyelocytic leukemia patients; first or subsequent relapse or refractory disease; adequate cardiac function (LVEF ≥50%); positivity of blast cells to the CD33 antigen.

The median age was 47 years (range 19–63). Twenty patients were females and 22 males. Thirteen patients had refractory disease, 24 patients were in 1st relapse (median 1st CR duration 11 months, range 1–95) and 5 patients had more advanced disease. One patient in 1st relapse had received an allograft in 1st CR and suffered the relapse 95 months after the 1st CR. Nine patients (22%) had high-risk cytogenetic features and 10 (25%) were FLT3 ITD+. Eleven of the 42 patients had received a stem cell transplant in 1st CR; more in particular, 8 patients had undergone an autologous transplant and 3 an allogeneic stem cell transplant.

The biological and clinical risk factors of the patients are detailed in Table 1.

### Salvage Regimen and Supportive Care

The chemotherapy regimen (AIM) consisted of intermediate dose of Ara-C (1 g/m^2^/day by four-hour intravenous infusion on days 1–5), Idarubicine (8 mg/m^2^/day by one hour intravenous infusion on days 1, 3 and 5), GO (3 mg/m^2^/day by two hours intravenous infusion on day 6) and Peg-filgrastim 6 mg fixed dose by subcutaneous injection on day 8.

Before GO administration, all patients received steroids (40 mg of methylprednisolone), antihistamine and acetaminophen 1g orally to prevent or ameliorate the infusion-related symptoms. Prophylactic oral quinolones were given when the neutrophil count fell below 0.5 × 10^9^/l. Acyclovir i.v. (250 mg/8h) was started on day +9 to prevent Herpes virus infection. Intravenous broad spectrum antibiotics were given for fever during aplasia. In case of persistence of fever over 72 hours after the beginning of antibiotic therapy, a work-up was performed to investigate the presence of a fungal infection (cranial, paranasal and thorax CT scan, evaluation of galactomannan serum levels and plugs). For patients who had experienced a fungal infection during previous treatments, antifungal therapy was given at the end of the chemotherapy regimen as secondary prophylactic treatment. All blood products were irradiated prior to infusion to prevent graft-versus-host reactions. Low-dose heparin for veno-occlusive disease (VOD) prophylaxis was not scheduled in our patients.

### Response Criteria

A CR was defined as a cellular bone marrow with less than 5% of blasts, a normalization of peripheral blood counts and a disappearance of all clinical features related to leukemia persisting for more than one month. A patient was defined as refractory if either progression of disease during induction or a persistent bone marrow blastosis on day +28 from the start of chemotherapy were recorded. Relapse was defined as a reappearance of leukemic blasts in the peripheral blood or ≥5% blasts in the bone marrow in two consecutive bone marrow aspirates performed at a one week interval.^14^

### Statistical Analysis

Data were analyzed as of June 2012. Overall survival (OS) was calculated from the start of chemotherapy to the date of death from any cause or of the last follow-up. Disease-free-survival (DFS) was calculated from the date of CR to the date of relapse or death and to the last follow up for patients in CR. OS and DFS probabilities were estimated using the Kaplan-Meier method as calculated according to the SPSS software.

## Results

The overall CR rate was 76% (32/42 patients). The median time to remission was 27 days (range 16–94). Two patients achieved a CR after two induction cycles. Ten patients (24%) proved refractory, but only 5/10 belonged to a high-risk category (4 FLT3 ITD+, 1 secondary leukemia). No induction-related deaths were reported. The results are shown in Table 2.

**Figure 1:**
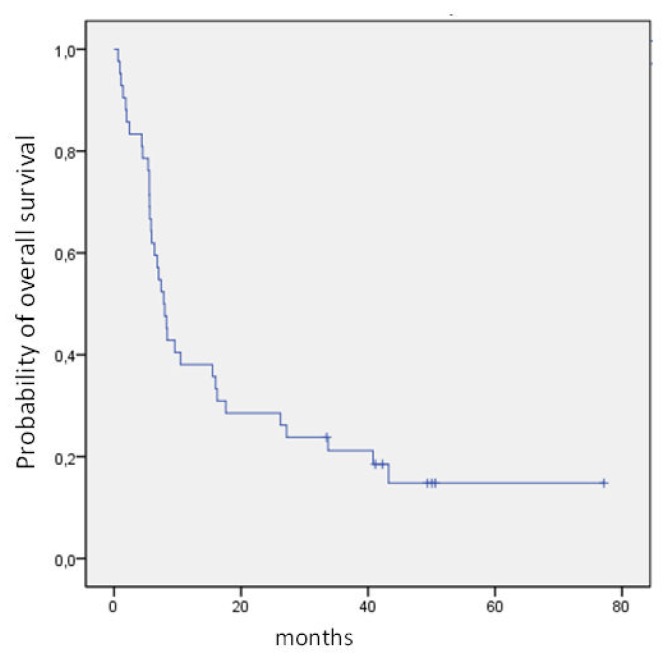
Kaplan Meyer estimate of overall survival

**Figure 2:**
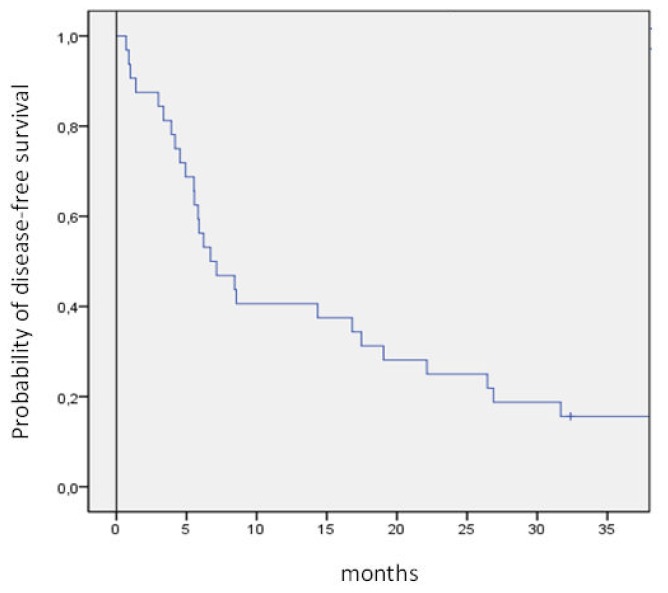
Kaplan Meyer estimate of disease-free survival

The median OS and disease-free survival (DFS) times are 8 months (range 5–50) and 7 months (range 1–50), respectively. The two-year OS and DFS rates are 29% and 25%, respectively (Fig. 1, Fig. 2).The cumulative rates of relapse are 41% and 45% at 1 and 2 years, respectively. Among the 32 responding patients 25 have died. The cause of death was AML in 12 patients, infection in 1 patient and transplant-related toxicity in 12 patients. None of the transplant-related deaths was due to the occurrence of VOD.

By log rank analysis, consolidation treatment before transplant had no significant impact on survival (p: 0.5).

### Post-remission Therapy

Patients achieving a CR were submitted, as soon as possible, to a transplant procedure. In the case of a delay in donor availability, they received a consolidation course with the same chemotherapy schedule. Among the 32 patients who achieved a CR, 5 did not receive further chemotherapy after the AIM regimen because of early relapse (n=1), poor performance status (n=1) and planned donor lymphocyte infusion (DLI) (n=3), because of a previous allograft. Twenty-two patients received 1 or 2 consolidation cycles.

In 3 patients, chemotherapy was followed by an autograft. Fifteen patients could proceed to an allogeneic HSCT, directly after AIM induction (n=5) or after consolidation chemotherapy (n=10). Allogeneic stem cell transplant consisted of a standard matched related HSCT in 4 patients, a matched unrelated allogeneic HSCT in 7 patients, a haploidentical HSCT in 4.

### Toxicity

The median time required to attain an absolute neutrophil count in excess of 0.5 × 109/L was 19 days (range 16–50). A sustained platelet count exceeding 50 × 10^9^/L was reached after a median of 27 days (range 17–108). The median duration of neutropenia was 14 days (range 5–55). Extra-hematological toxicity was mild: 7 patients (17%) suffered a grade III-IV mucositis according to the WHO classification. No patient presented alopecia. Thirty patients had fever; in 20 of them (48%), a bacteremia was documented.

Out of 42 patients, 15 (36%) had experienced fungal infections during previous treatments and therefore received successful systemic prophylactic antifungal therapy during aplasia. Among the remaining patients, 2 developed a fungal infection: pneumonia in 1 case and a spleen abscess in the other.

Neither VOD nor other sign of hepatic toxicity occurred.

## Discussion

Although the possibility of achieving a CR in patients with AML has substantially improved over the last decade, relapsed/refractory AML has a very poor prognosis with standard chemotherapy; thus, the possibility of undergoing a transplant procedure has to be considered the goal for any re-induction therapy.^15^

Recently, the development of targeted strategies with compounds more specifically directed against leukemic cells, like the FLT3 inhibitors and the farnesyl-transferase inhibitors, have opened new possibilities for the management of relapsed and refractory AML. GO is the only compound for which there are a number of phase II/III studies in the literature.^9^–^13^ In the phase III MRC AML15 study, a single dose of GO at 3 mg/m^2^ during the first and third course of chemotherapy was associated with a significant survival benefit in the subset of patients with favourable cytogenetics. Recently the ALFA 0701 study showed that the addiction of fractionated doses of GO to standard chemotherapy (3/3/3 regimen) improves the survival outcome in patients aged 50–70 years with the novo AML. The authors conclude that the fractionated regimen allows the delivery of a high cumulative dose of GO without excessive toxicity.^16^,^17^ In our study, using a new salvage schedule combining chemotherapy with GO and Peg-filgrastim (AIM), we report a high overall CR rate (76%) in the high-risk group of relapsed/refractory AML patients, most of whom had already received a stem cell transplant in 1^st^ CR, had unfavorable molecular and cytogenetic markers, and a 1^st^ CR duration shorter than one year. Despite the relatively limited patient sample size, the remission rate recorded is comparable to that obtained in newly diagnosed AML patients.

The therapeutic regimen was well tolerated. No induction deaths were observed and the hematological toxicity proved comparable to that of other salvage schedules previously reported. The low fungal infection rate reported is related to a high prevalence of antifungal prophylaxis (36%) because of previous fungal infections. No patient developed a VOD or other hepatotoxic symptoms; this occurred both in patients who had received a transplant procedure in 1st CR and in those treated with azole antifungal agents, which can be considered as other potential factors contributing to liver toxicity. Gut toxicity was nearly absent and no patient presented alopecia. Possible explanations for this extremely mild morbidity may be the choice of utilizing an intermediate dose of Ara-C, which makes it feasible also in heavily pretreated patients, and the use of a reduced dose of GO at 3 mg/m^2^ based on its documented low toxicity profile.

The protocol design has several advantages: first of all, it combines two of the most efficient agents in AML, such as intermediate doses of Ara-C and Idarubicin, with the anti-CD33 antibody; secondly, GO is administered at the end of chemotherapy, after the debulking phase. Finally, the addition of Peg-filgrastim at the end of chemotherapy might enhance GO-induced apoptosis and inhibit P-gp expression in AML cells.^18^,^19^

Chevallier and colleagues reported their experience on 62 patients with primary resistant or relapsed AML treated with Mitoxantrone, intermediate-dose Ara-C and Gentuzumab Ozogamicin, documenting a 2-year overall survival of 41%.^20^ The main difference between the present study and the French one is that in the latter the proportion of patients achieving a response was 63%, and only 12/39 patients (30%) proceeded to an allogeneic transplant, which was in the majority of cases a reduced intensity conditioning transplant.

In recent years, the development of reduced intensity conditioning regimens has enabled older and more comorbid patients to undergo an allogeneic HSCT. These approaches rely more heavily on the potent graft-versus leukemia (GVL) effects for tumor eradication/control than on chemotherapy doses and are associated with survival rates between 28% and 54% also because of the low risk of morbidity and mortality.^21^

In our series, the median OS from relapse is 8 months with the 2-year projected OS and DFS of 29% and 25%, respectively. The relapse rate is similar to that reported in the literature, but 48% of deaths were related to transplant procedures either performed directly after treatment or after a subsequent relapse.

Unfortunately, due to the results reported by the SWOG Group in the phase III S0106 study,^22^ documenting an increased number of deaths during induction therapy in the GO group, GO has recently been withdrawn from the market in the US and Europe, and this has prevented us to increase the patient population. Nevertheless our results suggest that the AIM regimen is safe and feasible, capable of providing a high response rate in relapsed/refractory AML patients and of enabling a high proportion of patients to proceed to a transplant procedure. In this category of heavily pretreated patients, a non-myeloablative allogeneic transplant may contribute to improve the long-term survival probability.

## Figures and Tables

**Table 1:** 

PATIENTS’ CHARACTERISTICS		NO. OF PATIENTS	
		(TOTAL 42)	%
SEX (m/f)		22/20	52/47
MEDIAN AGE, YEARS (range)		47 (19–63)	
	≥60	4	9
	<60	38	90
CYTOGENETICS	FAVORABLE	1	2
	INTERMEDIATE	30	71
	ADVERSE	9	9
	UNKNOWN	2	4
FLT3 ITD		10	23
DISEASE PHASE	REFRACTORY	13	31
	> I RELAPSE	5	12
	I RELAPSE	24	57
I^ST^ CR DURATION	≤12 MONTHS	15/24	62
	>12 MONTHS	10/24	37
PREVIOUS TREATMENTs	PREVIOUS AUTOGRAFT	8/29	27
	PREVIOUS ALLOGRAFT	3/29	10

**Table 2:** 

OUTCOME		NO. OF PATIENTS	%
COMPLETE REMISSION	OVERALL	32/42	76
	PATIENTS IN RELAPSE	18/24	75
	REFRACTORY PATIENTS	10/13	69
	ADVANCED DISEASE	5/5	100
POSTREMISSION THERAPY	NONE	5/32	15
	CHEMOTHERAPY ALONE	9/32	28
	ALLOGRAFT	5/32	15
	CHEMOTHERAPY THEN AUTOGRAFT	3/32	9
	CHEMOTHERAPY THEN ALLOGRAFT	10/32	31
CAUSE OF DEATH	RELAPSE OR PROGRESSION	12/25	48
	BMT-RELATED TOXICITY	12/25	48
	INFECTION	1/25	4
